# Reciprocal relationships between self-esteem, coping styles and anxiety symptoms among adolescents: Between-person and within-person effects

**DOI:** 10.1192/j.eurpsy.2023.1573

**Published:** 2023-07-19

**Authors:** W. Li, C. Lu

**Affiliations:** 1 school of public health, Sun Yat-sen University, Guangzhou; 2 公共卫生学院, 中山大学, 广州, China

## Abstract

**Introduction:**

Previous researches have not distinguished between-person effects from within-person effects when exploring the relationship between self-esteem, coping styles, and anxiety symptoms among adolescents.

**Objectives:**

To address this gap, this study investigated reciprocal associations among self-esteem, coping styles, and anxiety symptoms in a three-wave longitudinal panel survey, using an analytical strategy that disaggregates within-person and between-person variance.

**Methods:**

The data was drawn from the Longitudinal Study of Adolescents’ Mental and Behavioral Well-being Research study conducted in 10 public schools in the Guangdong province of China. All participants had a baseline visit (N = 1957, age 13.6, grades 7 and 10) and follow-up interviews at one-year intervals for 3 years. A random intercept cross-lagged panel model combined with mediation analysis was performed.

**Results:**

At the within-person level, following results were observed. (1) Low self-esteem and anxiety symptoms bidirectionally predicted each other. (2) Low self-esteem and negative coping style bidirectionally predicted each other. (3) Anxiety symptoms predicted subsequent negative coping style but not vice versa. At the between-person level, we obtained the following main results. (1) Significant predictive effects on the random intercept were found among all three study constructs. (2) There were sex differences regarding the association between self-esteem and anxiety symptoms and the correlation strength of females was greater than that of males. (3) Self-esteem mediated the reciprocal relations between coping styles and anxiety symptoms.

**Conclusions:**

These results could be an important advance by elucidating the reciprocal relationships among self-esteem, coping styles, and anxiety symptoms at the within-person level, suggesting that interventions targeted at promoting self-esteem and cultivating positive coping style may help reduce adolescent anxiety.

**Disclosure of Interest:**

None Declared

